# Calibration-Free Pulse Oximetry Based on Two Wavelengths in the Infrared — A Preliminary Study

**DOI:** 10.3390/s140407420

**Published:** 2014-04-23

**Authors:** Meir Nitzan, Salman Noach, Elias Tobal, Yair Adar, Yaacov Miller, Eran Shalom, Shlomo Engelberg

**Affiliations:** 1 Department of Applied Physics/Medical Engineering, Jerusalem College of Technology, Jerusalem 9116001, Israel; E-Mails: salman@jct.ac.il (S.N.); elitobal@gmail.com (E.T.); yadar@g.jct.ac.il (Y.A.); yalamiller@gmail.com (Y.M.); eransh@g.jct.ac.il (E.S.); 2 Department of Electronics, Jerusalem College of Technology, Jerusalem 9116001, Israel; E-Mail: shlomoe@jct.ac.il

**Keywords:** oxygen saturation, pulse oximetry, infrared, arterial blood, diode lasers, calibration

## Abstract

The assessment of oxygen saturation in arterial blood by pulse oximetry (SpO_2_) is based on the different light absorption spectra for oxygenated and deoxygenated hemoglobin and the analysis of photoplethysmographic (PPG) signals acquired at two wavelengths. Commercial pulse oximeters use two wavelengths in the red and infrared regions which have different pathlengths and the relationship between the PPG-derived parameters and oxygen saturation in arterial blood is determined by means of an empirical calibration. This calibration results in an inherent error, and pulse oximetry thus has an error of about 4%, which is too high for some clinical problems. We present calibration-free pulse oximetry for measurement of SpO_2_, based on PPG pulses of two nearby wavelengths in the infrared. By neglecting the difference between the path-lengths of the two nearby wavelengths, SpO_2_ can be derived from the PPG parameters with no need for calibration. In the current study we used three laser diodes of wavelengths 780, 785 and 808 nm, with narrow spectral line-width. SaO_2_ was calculated by using each pair of PPG signals selected from the three wavelengths. In measurements on healthy subjects, SpO_2_ values, obtained by the 780–808 nm wavelength pair were found to be in the normal range. The measurement of SpO_2_ by two nearby wavelengths in the infrared with narrow line-width enables the assessment of SpO_2_ without calibration.

## 1. Introducation

### 1.1. Pulse Oximetry for the Measurement of Arterial Oxygen Saturation

The transfer of oxygen from the lungs to the tissue cells is largely carried out by the hemoglobin molecules in the red blood cells and only 2% of the total oxygen content is dissolved in the plasma. Oxygen saturation in the blood is the ratio of oxygenated hemoglobin concentration to total hemoglobin concentration in the blood, and its value in the arterial blood, SaO_2_, is of great clinical and physiological significance since it reflects the adequacy of oxygen delivery and respiratory function. Normal values of SaO_2_ are 94%–98% at sea level but the values may decline somewhat beyond the age of 70 years [1].

**Figure 1.**
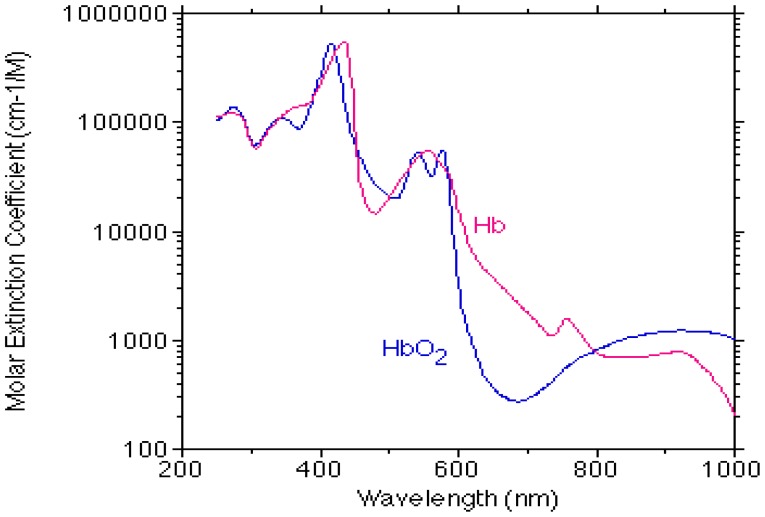
Molar extinction coefficients of oxygenated (HbO_2_) and deoxygenated (Hb) hemoglobin as a function of the wavelength. Prepared by Dr. Scott Prahl from a variety of sources [7]. Used with permission from Dr. Prahl.

**Figure 2.**
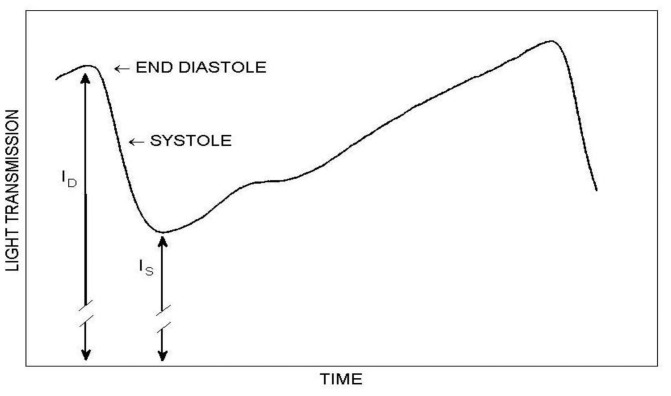
The PPG pulse. The transmitted light through the tissue decreases during systole and increases during diastole. I_D_ and I_S_ represent the maximal and minimal light transmission through the tissue.

SaO_2_ can be assessed *in vitro*, in extracted arterial blood, either directly by means of co-oximetry or by measuring oxygen partial pressure and using the oxygen-hemoglobin dissociation curve. Estimation of SaO_2_ can also be obtained non-invasively by pulse oximetry [2–4], which is based on the different light absorption spectra for oxygenated and de-oxygenated hemoglobin (Figure 1). In order to assess SaO_2_, the contribution of the arterial blood to the light absorption must be isolated from that of the venous blood, and in pulse oximetry it is achieved by photoplethysmography (PPG)–the measurement of light absorption changes due to the cardiac-induced blood volume changes. The PPG probe consists of a light source emitting light into the tissue and a detector measuring the intensity of light transmitted through the tissue, which decreases during systole because of the systolic increase in the arterial blood volume (Figure 2). Since the PPG pulse represents light absorption in arterial blood, PPG signals at two wavelengths enable the assessment of oxygen saturation in the arterial blood [2]. As will be explained below, there is still a discrepancy between the value of arterial oxygen saturation obtained by pulse oximetry and that obtained by direct measurements in blood extracted from the arteries, and it is therefore customary to designate the former by SpO_2_ while the latter retains the name SaO_2_. [4–6].

The theory of conventional pulse oximetry has been described in several publications [3,8–10]. PPG curves in two wavelengths are recorded and SpO_2_ is derived from the ratio of ratios R which is defined by:

(1) $$R=\frac{{\left(AC/DC\right)}_{1}}{{\left(AC/DC\right)}_{2}}$$

where AC and DC are the peak-to-peak amplitude and the baseline of the PPG pulse, respectively. In commercial pulse oximeters the two wavelengths are chosen in the red and infrared regions, where the difference in *light absorption* between the two wavelengths is relatively large. However, the transmitted light intensity through a tissue sample which includes blood vessels is also affected by *light scattering* in the tissue and in the blood. For the choice of two wavelengths in the red and infrared the scattering coefficient and the optical path-length differ significantly between the two wavelengths [3,9,11,12] and the relationship between the physiological parameter, SaO_2_, and the measured parameter R cannot be derived directly from physical and physiological considerations of light absorption in oxygenated and deoxygenated hemoglobin, using the Lambert-Beer Law. The relationship between R and SaO_2_ is found experimentally for each type of commercial pulse oximeter sensor by calibration [3,4]: R is measured in several persons simultaneously with *in vitro* SaO_2_ measurement in extracted arterial blood by means of a co-oximeter. The formula relating R to oxygen saturation measured by pulse oximetry, SpO_2_, is then determined by proposing a mathematical relationship, such as:

(2) $${\text{SpO}}_{2}=\frac{{\text{k}}_{1}-{\text{k}}_{2}\text{R}}{{\text{k}}_{3}-{\text{k}}_{4}\text{R}}$$

and obtaining the values of the constants k_i_ for the specific pulse oximeter by best fit analysis of the measured parameters in the calibration process.

An analytical relationship between R and SpO_2_ can be obtained by making use of the Lambert-Beer Law and analysis of the light absorption and scattering in tissue which includes blood with oxygenated and deoxygenated hemoglobin [9,11]. The following relationship between the ratio R and the oxygen saturation SpO_2_ is then derived:

(3) $${\text{SpO}}_{2}=\frac{{ɛ}_{\text{d}1}-\text{R}({\text{l}}_{2}/{\text{l}}_{1}){ɛ}_{\text{d}2}}{\text{R}({\text{l}}_{2}/{\text{l}}_{1})({ɛ}_{02}-{ɛ}_{\text{d}2})+({ɛ}_{\text{d}1}-{ɛ}_{01})}(3)$$

where ε_o_ and ε_d_ are the extinction coefficients for oxygenated and deoxygenated hemoglobin, respectively (the extinction coefficient of hemoglobin is defined as the absorption constant of the sample divided by the hemoglobin concentration). The constants l_1_ and l_2_ are the path-lengths for the two wavelengths and depend strongly on the scattering coefficient.

For the two wavelengths in the red and infrared regions which are used by the common commercial pulse oximeters, l_1_ and l_2_ are expected to differ and they are unknown. SpO_2_ can be derived from R through the calibration process by assuming that l_2_/l_1_ is a constant that is independent of inter-subject variability in the circulatory system. In this case the coefficients of R in Equation (3) are constants and can be determined through calibration, as described above [5,11]. If the parameter l_2_/l_1_ changes between different subjects, in particular between the healthy subjects on whose fingers the calibration was performed and the patients on whose fingers the clinical examination is carried out, inaccuracy in SpO_2_ measurement is to be expected.

### 1.2. Accuracy of Pulse Oximetry

Most manufacturers of pulse oximeters claim an accuracy of 2% [13,14], which is the standard deviation (SD) of the differences between SpO_2_ and SaO_2_ measured on extracted arterial blood samples by a co-oximeter. An SD of 2% means that an error of 4% or more (2SD) is expected in 5% of the examinations, assuming that the differences between SpO_2_ and SaO_2_ are normally distributed. Since the full range of SaO_2_ in adults, even including most of the sick patients is 80%–100%, an error of 3%–4% may be significant.

Despite its potential error of 3%–4%, pulse oximetry is a clinically valuable tool for monitoring patients during surgery or rehabilitation, ensuring intraoperative and post-operative patient safety. Pulse oximetry can detect a sudden decrease of SpO_2_ by 3%–4%, enabling the early detection of acute deterioration of the respiratory function of the patient or technical failure of mechanical ventilatory support and is, therefore, considered a mandatory tool for most patients in operating rooms and intensive care units.

An inaccuracy of 3%–4% in oxygen saturation measurement is, however, too high to allow such pulse oximeters to be used in routine management of various patient populations, in particular critically ill patients. Because of the low accuracy of SpO_2_ measurements, some researchers suggested that SpO_2_ levels as high as 94% or 96% should be maintained, in order to ensure SaO_2_ value of 90% during mechanical ventilation and oxygen support [1,15,16]. In a study on critically ill patients, relatively low correlation was found between *spontaneous changes* in SpO_2_ and in SaO_2_ (r = 0.6, r^2^ = 0.37) [17], and the authors inferred that changes in SpO_2_ do not reliably predict equivalent changes in SaO_2_ in the critically ill. In a study on patients with severe sepsis and septic shock in emergency department, the mean difference between SpO_2_ and SaO_2_ was 2.75% and the standard deviation 3.1% [18].

The low accuracy of pulse oximetry can be attributed, at least partly, to the calibration process currently used for pulse oximeters, which is based on examinations on healthy volunteers and is not necessarily applicable to sick patients, in particular neonates [14]. Since the calibration process is based on SaO_2_ measurements by co-oximeter, the inaccuracy in the latter also contributes to the error in SpO_2_ measurement [13]. The discrepancy between SpO_2_ and SaO_2_ is greater at saturations below 75%–85% [1,10,18–20], because ethical restrictions prevent manufacturers from reducing SaO_2_ below 80% during the calibration process.

### 1.3. A Calibration-Free Pulse Oximeter

If the two wavelengths are sufficiently close so that the difference between their path-lengths can be neglected (l_2_/l_1_ ∼ 1), the relationship between the ratio R and SpO_2_ becomes [3,8,21]:

(4) $${\text{SpO}}_{2}=\frac{{ɛ}_{\text{d}1}-\text{R}{ɛ}_{\text{d}2}}{\text{R}({ɛ}_{02}-{ɛ}_{\text{d}2})+({ɛ}_{\text{d}1}-{ɛ}_{01})}$$

Equation (4) enables the calculation of SpO_2_ from the measured parameter R and the values of the extinction coefficients with no need for calibration. This was shown by Nitzan *et al.* [21], using two infrared light emitting diodes (LEDs) with emission spectra that peaked at wavelengths 767 and 811 nm. The SpO_2_ values, found using Equation (4), were in the range of 90%–100%, while SpO_2_ values obtained by commercial pulse oximeters (using red and infrared light and calibration) were 96%–98%. In that study, the values of the hemoglobin extinction coefficients in Equation (4) were taken as the mean values of the corresponding values presented in several data bases, averaged over the emission spectra of the LEDs.

The low accuracy achieved by the calibration-free pulse oximetry in that study [21] can be attributed, at least partly, to the broad emission spectra of the LEDs resulting in inaccuracy in the determination of the mean extinction coefficient. In the current study, the LEDs, have been replaced by infrared laser diodes. The narrow emission spectra of the laser diodes allow the use of specific values of the extinction coefficients for hemoglobin. Additionally, as described in the next section, the analysis of the PPG pulses was improved.

## 2. Experimental Section

### 2.1. Subjects and Methods

SpO_2_ was measured on 15 male subjects aged 20–35 years. Each finger temperature was above 30 °C. For each subject PPG signals in three wavelengths, 780, 785 and 808 nm, were recorded for about 90 s, and SpO_2_ was derived from each pair of wavelengths as described below. Simultaneous SaO_2_ measurements were also performed on these subjects using a commercial pulse oximeter (BCI 3301 Smiths Medical PM, Waukesha, WI, USA**)** for comparison.

### 2.2. The Optical System

When using Equation (4) to determine the value of SpO_2_, it is important to ensure that the wavelength of the light used to measure the PPG is stable. An error of ±0.2 nm in the wavelength causes an error in the determination of the extinction coefficient and leads to an error of about ±0.5% in SaO_2_ for the 780 nm–808 nm and 785 nm–808 nm pairs and much more for the 780 nm–785 nm pair. Hence, a well-defined, stable wavelength is needed for the calibration-free pulse oximetry.

In the current study we used three wavelength-stabilized laser diodes (Innovative Photonic Solutions, Inc. Monmouth Junction, NJ, USA) of wavelengths 780, 785 and 808 nm, with narrow spectral bandwidth (less than 0.15 nm). For each diode, the manufacturer's data sheets were used to determine a temperature range over which the wavelength dependence on temperature is small. Thermoelectric coolers (TECs) (TCLDM9 Thorlabs, Newton, NJ, USA) that make use of the Peltier effect were used to stabilize the temperature (mainly in order to stabilize the light intensity). Changes in the TEC temperature by ±3 °C and changes in the diode current in the range 80–140 mA changed the wavelength by less than 0.1 nm (measured by an Agilent 86140, optical spectral analyzer, Santa Rosa, CA, USA).

**Figure 3.**
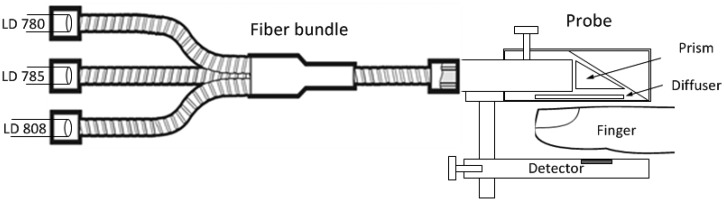
Schematics of the optical setup. The three laser diodes were coupled to a trifurcated fiber bundle. The common bundle was attached to the finger probe parallel to the finger surface. The reflecting prism is opposite to the detector.

The light emitted from each of the three light sources needs to be delivered to the same site in the finger in order to illuminate the same blood volume for each pair of wavelengths. The beams from the three laser diodes were merged randomly and delivered to the same measurement site by using a trifurcated fiber bundle (Oriel-Newport Model 77536, Irvine, CA, USA) which was comprised of three input bundles (each with a diameter of 3.2 mm) and a common bundle (with a diameter of 5.5 mm) (Figure 3). The latter was comb randomized, so the output light from each input bundle was evenly divided in the common bundle.

**Figure 4.**
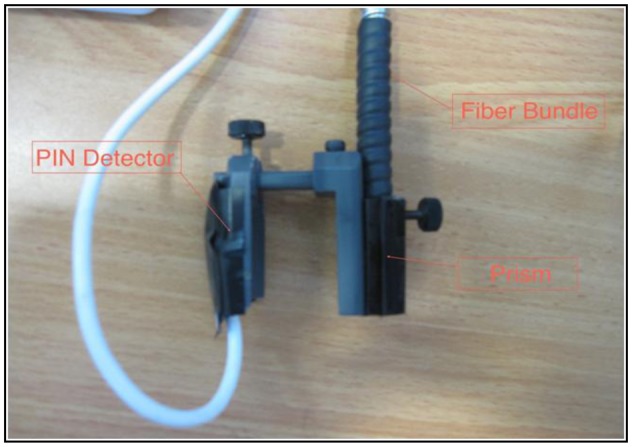
Photograph of the finger probe. Light is delivered to one side of the finger and the transmitted light is collected by the detector.

The finger transmission probe (Figure 3) had an input port for the common fiber bundle end and a PIN photodiode (BPW 34, Vishay Electronics, Malvern, PA, USA, 5.4 × 4.3 mm^2^) placed opposite the fiber bundle end. The light was reflected by 90° through a prism inside the probe, allowing the fiber bundle to be attached parallel to the finger, for higher mechanical stability. A diffuser (Light Shaping Diffuser, Luminit, Torrance, CA, USA) was placed after the prism to homogenize the light distribution and to reduce speckle noise. A photograph of the probe is shown in Figure 4.

### 2.3. The Electronic System

**Figure 5.**
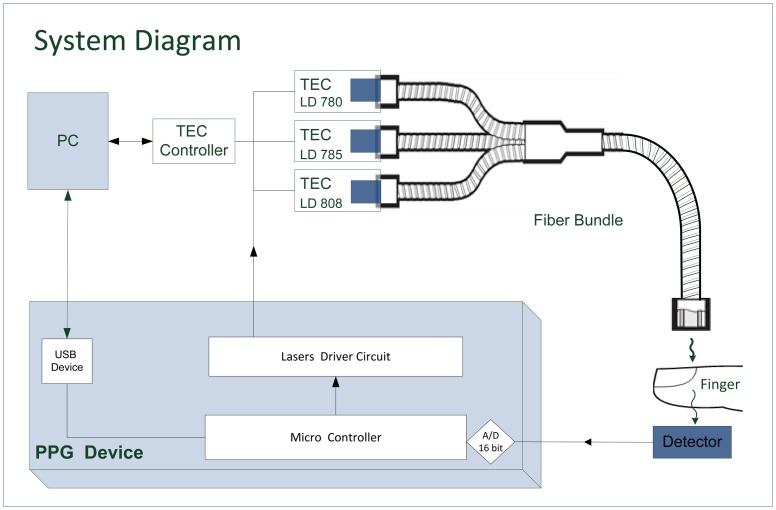
Block diagram of the measurement system.

A block diagram of the measurement system is given in Figure 5. Light from the laser diodes is emitted sequentially, by time sharing, using driver circuits controlled by a microcontroller. The light transmitted through the finger is detected by the photodetector, whose output is digitized and processed by the microcontroller and conveyed to a PC for offline analysis. The temperature of each TEC is determined via the PC. The power to the PPG device was supplied by three 1.5 V AA batteries.

The PPG signal for each wavelength was separated by means of time sharing between the laser diodes. During each cycle of 2,556 μs, each laser diode was turned on for 511.2 μs and off for the rest of the cycle. Each of the three wavelengths and the dark current were sampled 391.22 times per second (in order to achieve good temporal resolution for the PPG pulses at the three wavelengths and to avoid aliasing of 50 Hz noise). Consequently, the detector output was sampled at 1564.88 Hz and digitized using a 16-bit A/D.

**Figure 6.**
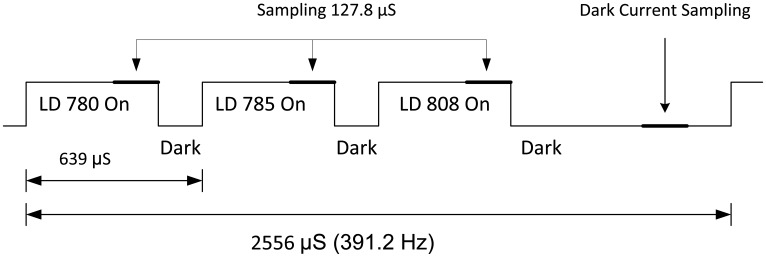
Schematic presentation of the time division of the laser diode activation between the three diodes.

The first sample of the detector output for each laser diode was taken 383.4 μs after turning on the laser diode. This was done in order to stabilize the laser diode, so that the actual sampling was carried out for a period of 127.8 μs (Figure 6). During this interval, 25 readings were acquired by the A/D and averaged by the microcontroller. Then a dark period of 127.8 μs was used to avoid cross-talk between channels. The second and third laser diodes were pulsed in the same sequence, and afterwards the dark current was sampled. Figure 6 shows a diagram of the time division. The signal was exported to MATLAB for post-processing and the system had the ability to display the signal in real time on the PC.

### 2.4. Determination of SpO_2_

**Figure 7.**
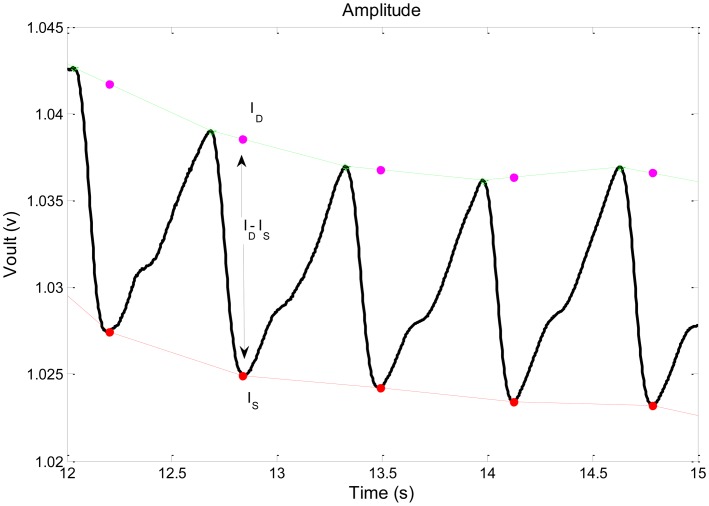
The extrapolated values of I_D_, and the determination of the pulse amplitude as the difference between the extrapolated values of I_D_ and the measured value of I_S_.

SpO_2_ was determined by using Equation (4). The analytical derivation of Equation (4) from the Beer–Lambert law [11,21] shows that in order to obtain SpO_2_ through Equation (4) the ratio of ratios, R, should be calculated as follows:

(5) $$R=\frac{{\left[(I_{D}-I_{s})/I_{s}\right]}_{1}}{{\left[(I_{D}-I_{s})/I_{s}\right]}_{2}}$$

where I_D_ is the value of the PPG pulse at end-diastole and I_S_ is the minimum value of the PPG pulse during systole (see Figure 2). Note that the value of DC in Equation (1) was replaced by I_S_. Due to the slow trend of the baseline I_D_ of the PPG pulse, the value of I_D_ (for the determination of the pulse amplitude, I_D_–I_S_) was taken as the value of the extrapolated line connecting the two maxima of the pulse at the time of pulse minimum, as shown in Figure 7. The pulse amplitude was taken as the difference between the extrapolated value of I_D_ and the measured value of I_S_. For each wavelength, the pulse amplitude and I_S_ were derived for each of twenty pulses; for each pair of wavelengths the R value was calculated for each pulse by using Equation (5); then the mean value of R for the twenty pulses was taken as the R value for this pair of wavelengths.

In order to use Equation (4) to derive SaO_2_ the values of ε_o_ and ε_d_, the extinction coefficient values for oxygenated (HbO_2_) and deoxygenated (Hb) hemoglobin, are required for each wavelength. The absorption spectra for Hb and HbO_2_ have been measured and reported by several research groups [22–24], but the reported values differ between these groups [25]. Even a small change in the Hb and HbO_2_ extinction coefficients values can cause significant change in the SaO_2_ calculation when using Equation (4), since the latter includes differences between Hb and HbO_2_ extinction coefficients values in the infrared region, where these differences are small relative to those in the red region (Figure 1).

**Figure 8.**
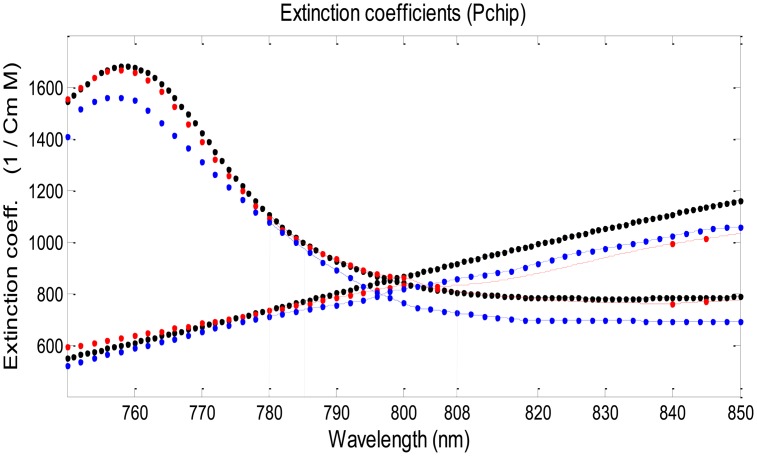
The extinction coefficients of Hb and HbO_2_ in the near infrared region as obtained by Zijlstra *et al.* (red), Cope (blue) and Prahl (Black) [25]. The red lines represent values derived from the experimental measurements of Zijlstra by means of interpolation (using the pchip command in MATLAB).

Tables of Hb and HbO_2_ extinction coefficients values are available from three research groups (Zijlstra [22], Cope [23] and Prahl [24]), and were also presented for analysis by Kim and Liu [25]. Figure 8 shows the extinction coefficients of Hb and HbO_2_ in the near infrared region of 750–850 nm as determined by the three groups. The data presented by Zijlstra in the region above 800 nm include only two points, at 805 and 840 nm, and interpolation was performed to determine values of the Hb and HbO_2_ extinction coefficients for 808 nm.

Table 1 presents the extinction coefficients for HB and HbO_2_ as derived from the tables of Zijlstra *et al.*, Cope and Prahl, for the three wavelengths which were used for the determination of SaO_2_ in the current study.

## 3. Results and Discussion

### 3.1. Results

PPG recordings for the three wavelengths, 780, 785 and 808 nm were performed on each of 15 healthy male volunteers. For each pair of wavelengths, the parameter R was calculated from the corresponding signals (using Equation (5)) and SaO_2_ was calculated using Equation (4), by making use of the corresponding values of the extinction coefficient from Table 1. In order to validate that the subjects had normal value of oxygen saturation, simultaneous measurements of SpO_2_ were made on them by means of the commercial pulse oximeter.

**Figure 9.**
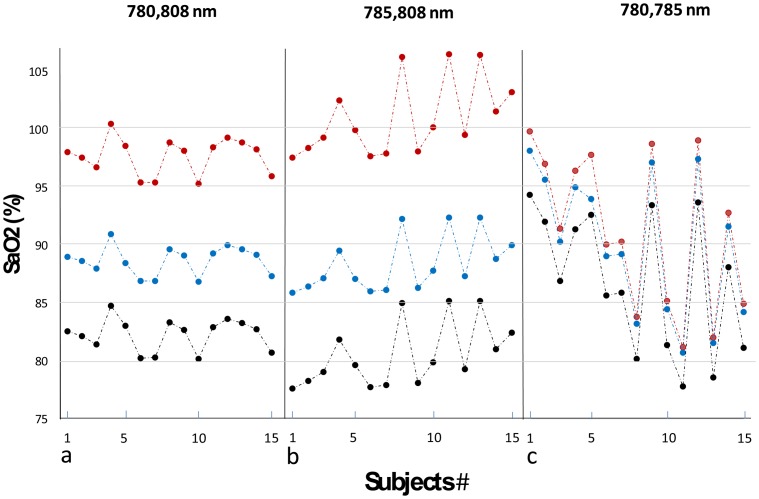
SpO_2_ values for the three pairs of wavelengths, determined by using the extinction coefficient data obtained by Zijlstra (red) Prahl (black) and Cope (blue).

Figure 9a shows SpO_2_ values as obtained for the pair 780–808 nm. The SpO_2_ values which were determined through the extinction coefficient values obtained by Zijlstra were between 95.3%–100.5%. These SpO_2_ values were similar to those obtained by the commercial pulse oximeter; the difference between them for each examinee was 2.5% or less (See Table 2). For this pair of wavelengths, SpO_2_ values determined through the extinction coefficient data obtained by Prahl and Cope were between 86.8%–91.0% and 80.2%–84.7%, respectively.

Figure 9b shows SpO_2_ values as obtained for the pair 785–808 nm. For this pair of wavelengths SpO_2_ values determined through the extinction coefficient values obtained by Zijlstra were between 97.5%–106.4%. For the extinction coefficient values obtained by Prahl and Cope SpO_2_ values were in the range 85.8%–92.4% and 77.6%–85.1%, respectively. Figure 9c shows SpO_2_ values as obtained for the 780-785 nm pair. For this pair of wavelengths SpO_2_ values determined by using the extinction coefficient values obtained by Zijlstra were between 81.2%–99.7% and for those obtained by Prahl and Cope were 0.5%–3.8% and 3.3%–5.4% lower, respectively.

The difference between the SpO_2_ values obtained for the three extinction coefficient databases was small for the wavelengths pair 780–785 nm (Figure 9c), relative to the other wavelengths pairs, because the difference in the extinction coefficient values between the three databases is relatively small for the wavelengths 780 and 785 nm while for 808 nm is larger (see Figure 8). Nevertheless, the small difference between the two wavelengths 780 and 785 nm relative to the other pairs of wavelengths and the fact that they lie on the same side of the isosbestic point lead to a large error in the determination of R and consequently of SpO_2_. Since R is quite close to 1 (R is equal to 1 for two equal wavelengths) and the values of the extinction coefficients for oxy- and deoxyhemoglobin are close, the values of the numerator and the denominator in Equation (4) are small, and a small error in the determination of R can cause a large error in the calculated value of SpO_2_.

### 3.2. Discussion

In the current preliminary study, we present a calibration-free pulse oximeter that makes use of two nearby wavelengths in the infrared region, assuming that the difference between their path-lengths can be neglected. In this case, by using the published data that gives the relevant extinction coefficients, SaO_2_ can be derived directly from Equation (4) without calibration. When the extinction coefficients were taken from the data of Zijlstra, SpO_2_ measurements, obtained by the calibration-free pulse oximeter, provided values in the normal range of oxygen saturation. It should be emphasized that we did not show that pulse oximetry which uses two wavelengths in the infrared is more accurate than conventional pulse oximetry; we did show that the former can measure SaO_2_ values in the normal range with reasonable accuracy. While the calibration process cannot decrease the error of conventional pulse oximetry to a level below 3%–4%, pulse oximetry with no need for calibration may have the potential to provide more accurate measurements of SaO_2_ than those provided by the currently available technique.

The proposed technique has several limitations:

(1). The small difference between the two wavelengths makes the values of the extinction coefficients quite close and causes the calculation of SpO_2_ to be very sensitive to the measured value of R. In order to measure SpO_2_ accurately, a very accurate measurement of the PPG pulse amplitude is required.
  In the current study three pairs of wavelengths were used, and only one of them, the 780–808 nm pair provided SpO_2_ results in the required range of oxygen saturation (when Zijlstra extinction coefficients values were used). The results of the 785–808 nm pair were of lower accuracy and the 780–785 nm pair showed the least accuracy. The correlation between the wavelength pair difference and the error in SpO_2_ measurement can be attributed to the greater sensitivity of SpO_2_ measurement to the accuracy of R measurement when two wavelengths with smaller difference between them are used.
(2). There are several databases in which values of the extinction coefficients for oxy- and deoxyhemoglobin have been reported, and there are significant discrepancies between the values obtained in the different studies. It seems that the main problem with the accurate determination of the extinction coefficients for oxy- and deoxyhemoglobin is the measurement of light attenuation in hemolized blood (hemolized blood is used in order to avoid the scattering of light by the red blood cells). The biochemical process of hemolization and the removal of the cells' remains probably interfere with the measurement of light absorption by the hemoglobin molecules. In the current study SpO_2_ results that lie within the normal range of oxygen saturation values were found only when the extinction coefficients values were selected from the data of Zijlstra; the use of Cope and Prahl data resulted in clearly erroneous values of SpO_2_. The accuracy of the calibration-free pulse oximetry depends on the accuracy of the extinction coefficients values, and additional research is required in order to find accurate values of the hemoglobin extinction coefficients.

The relative accuracy of the conventional and the calibration-free pulse oximetry should be determined through comparison with invasive *in vitro* measurements of SaO_2_. If the novel calibration-free pulse oximetry is found to be more accurate than the conventional method, it should have additional diagnostic value in several clinical applications.

It should be noted that some other approaches on calibration-free SpO_2_ measurement have been proposed in the literature. Reddy *et al.* [26] suggested a method based on a mathematical model for the attenuation of light passing through the finger. According to their method SpO_2_ is derived from the amplitudes and slopes of the PPG waveforms acquired at red and infrared excitation wavelengths and the extinction coefficients for Hb and HbO_2_. Preliminary examinations performed on healthy volunteers and patients showed agreement with a commercial pulse oximeter. Another calibration-free method based on frequency-modulated near infrared spectroscopy (NIRS) was suggested by Franceschini *et al.* [27]. However both techniques are based on mathematical models that match the tissue circulation only in approximate terms.

## 4. Conclusions

The pulse oximetry technique for the measurement of oxygen saturation in arterial blood has been improved significantly since its emergence about 30 years ago, but it is still not accurate to better than three or four percent. This inaccuracy is due, at least in part, to the calibration process used, while this process is essential when two significantly different wavelengths are used. The great success of pulse oximeters in detecting significant decrease of oxygen delivery and deterioration of respiratory function in patients during surgical procedures and during recovery probably masks the fact that the accuracy of the present technique is not sufficient to allow its reliable use in certain medical problems, in particular SaO_2_ monitoring during oxygen supplementation for critically ill patients. Pulse oximetry which uses two nearby wavelengths is a greater technological challenge, but since it does not require initial calibration it has the potential to provide the clinical community with more accurate arterial oxygen saturation values than those obtained by the available pulse oximeters which use two wavelengths in the red and infrared regions and need calibration.

## Figures and Tables

**Table 1.** The extinction coefficients for HbO_2_ and Hb, ε_o_ and ε_d_, respectively, as derived from the tables of Zijlstra *et al*, Cope and Prahl.

**Wavelength [nm]**	**ε_o_/ε_d_[1/cm M]**		

	**Cope**	**Prahl**	**Zijlstra**
780	738/1,098	712/1070	733/1085
785	769/994	736/973	757/992
808	918/803	857/723	831/806

**Table 2.** SpO_2_ values, obtained by the calibration-free pulse oximetry (based on the 780–808 nm wavelength pair and on the Zijlstra data) and the commercial pulse oximeter for the 15 healthy subjects.

**#**	**Calibration-Free (%)**	**Commercial (%)**
1	98.1	97
2	97.6	96
3	96.7	98
4	100.5	98
5	98.6	97
6	95.4	96
7	95.4	97
8	98.9	97
9	98.2	97
10	95.3	96
11	98.4	97
12	99.3	98
13	98.9	97
14	98.3	97
15	95.9	96

## Notes

**Author Contributions:** Conception and design of the study: M. Nitzan. Design of the pulse oximeter and technical support: S. Noach, E. Tobal, Y. Adar, Y. Miller, E. Shalom. Collection of data: E. Tobal, Y. Adar, Y. Miller. Analysis and interpretation of the data: M. Nitzan, S. Engelberg, E. Tobal, Y. Adar. Manuscript preparation: M. Nitzan. Critical revision of the article: S. Engelberg.
